# Is the Effect of Parental Education on Offspring Biased or Moderated by Genotype?

**DOI:** 10.15195/v2.a6

**Published:** 2015-02-25

**Authors:** Dalton Conley, Benjamin W. Domingue, David Cesarini, Christopher Dawes, Cornelius A. Rietveld, Jason D. Boardman

**Affiliations:** a New York University; b University of Colorado, Boulder; c Erasmus University

**Keywords:** status attainment, genotype, gene-by-environment, parental education, heritability

## Abstract

Parental education is the strongest measured predictor of offspring education, and thus many scholars see the parent–child correlation in educational attainment as an important measure of social mobility. But if social changes or policy interventions are going to have dynastic effects, we need to know what accounts for this intergenerational association, that is, whether it is primarily environmental or genetic in origin. Thus, to understand whether the estimated social influence of parental education on offspring education is biased owing to genetic inheritance (or moderated by it), we exploit the findings from a recent large genome-wide association study of educational attainment to construct a genetic score designed to predict educational attainment. Using data from two independent samples, we find that our genetic score significantly predicts years of schooling in both between-family and within-family analyses. We report three findings that should be of interest to scholars in the stratification and education fields. First, raw parent–child correlations in education may reflect one-sixth genetic transmission and five-sixths social inheritance. Second, conditional on a child’s genetic score, a parental genetic score has no statistically significant relationship to the child’s educational attainment. Third, the effects of offspring genotype do not seem to be moderated by measured sociodemographic variables at the parental level (but parent–child genetic interaction effects are significant). These results are consistent with the existence of two separate systems of ascription: genetic inheritance (a random lottery within families) and social inheritance (across-family ascription). We caution, however, that at the presently attainable levels of explanatory power, these results are preliminary and may change when better-powered genetic risk scores are developed.

If researchers studying educational attainment merely want to describe the extent to which children resemble parents on this dimension of stratification, they need not concern themselves with the mechanisms by which such an intergenerational correlation is obtained. However, if scholars seek to explain how this social fact comes into being and, furthermore, wish to know whether policies that affect the distribution of education in one generation will have distributional consequences in the next generation, then whether the observed parent–child correlation in education reflects social inheritance or genetic inheritance should be of utmost importance. If family resemblance in educational attainment is socially oriented, then changes in the distribution of education in one generation will have important implications for the distribution of education in the next (holding fertility constant—an important factor in how educational changes present dynastic effects [Mare and Maralani 2006; Mare 1997]). However, if the intergenerational process is primarily due to the transmission of genes related to educational outcomes, then policies to equalize education would need to be applied continually (i.e., across generations) as long as the distribution of underlying genotype and the genotype–phenotype relationship hold steady. Furthermore, the extent to which the distribution of education is related to genetics and not environmental inputs may also have important implications for understanding other phenomena, such as income, health and happiness, or return to schooling. In short, even if researchers are uninterested in the genetic architecture of educational attainment, it is still important to know how the genotype–education relationship may bias the estimation of purely social models.

To provide additional motivation for studying the omnibus genetic influence on stratification outcomes such as education or income (i.e., heritability), some sociologists have persuasively argued that we should abandon raw or adjusted mobility rates (or intergenerational earnings elasticities) as measures of openness and meritocracy. Rather, Guo and Stearns (2002) and Nielsen (2006, 2008), among others, argue that we should compare the genetic component to the common environmental component of social status as determined by twin and other kin-based variance decomposition models. In this paradigm, it is not the *overall* correlation between siblings, for instance, that measures the relative openness or closure of a stratification system (cf. Björklund, Eriksson, and Jäntti 2002; Corcoran et al. 1992; Hauser and Sewell 1986; Hauser, Sheridan, and Warren 1999; Kuo and Hauser 1995; Olneck 1976; Page and Solon 2003; Warren and Hauser 1997; Warren, Sheridan, and Hauser 2002) but rather the proportion of that correlation that is due to shared genotype. That is, fundamentally unjust societies are evidenced by low heritability estimates where the genetic potential of the population is not fully realized because social factors are primarily responsible for phenotypic variation (Turkheimer et al. 2003). In this view, a meritocratic society would display a high genetic component to achieved social position and a low common (read: familial) environmental component. According to this argument, policy should aim to enhance sorting on innate characteristics and not the social advantages or disadvantages that may be conferred on us by our conditions of birth and upbringing (Heath et al. 1985).^1^

With these concerns in mind, ascertaining the proportion of a quantitative trait—such as IQ, education, or income—that is due to genetic variation has long been of interest to a wide range of social and behavioral scientists, despite the controversy surrounding such estimates (e.g., see Breen, Plomin, and Wardle 2006; Plomin, Owen, and McGuffin 1994, 1997; Plomin and Spinath 2004; Plomin 2009; Purcell 2002; Rodgers, Rowe, and Buster 1999; Rodgers, Buster, and Rowe 2001). Among human populations where experimentation is not possible, the workhorse of such analyses has been the twin or extended twin design, where the average relatedness of various kin pairs is correlated with their phenotypic similarity to ascertain the effect of shared genotype on a given outcome (Zaitlen et al. 2013). The reigning critique of this approach is that it is difficult to eliminate the possibility that increased similarity between, say, monozygotic twins as compared to dizygotic twins is due to more similar (exogenous) environments and not just their greater genetic similarity (Goldberger 1978, 1979; for a defense, see Barnes et al. 2014; Conley et al. 2013; Scarr and Carter-Saltzman 1979).

A recent meta-analysis that specifically examines the heritability of educational attainment across 36 different cohorts finds a heritability of ~40 percent, though there is significant variation among the individual studies (Branigan, McCallum, and Freese 2013). For example, a study of Italian twins finds a heritability of ~50 percent (Lucchini, Della Bella, and Pisati 2013). However, another recent paper uses U.S. data from the National Longitudinal Survey of Adolescent Health and, after accounting for assortative mating, obtains a genetic component of educational attainment of just under a quarter (Nielsen and Roos 2011). Sacerdote used a data set of Korean adoptees in the United States where assignment to families was random to examine the intergenerational correlation on important socioeconomic indicators such as educational attainment and income. Education (specifically probability of graduating from a four-year college) and income were inherited more strongly by biological children than by adopted children. However, the inheritance of health-related behaviors was similar across the two groups.

One of the most important limitations of this research is that genetic (and environmental) contributions to offspring educational attainment remain unmeasured; genotype—a measured variable—is not included. As such, we cannot directly assess each component (i.e., genetic and environmental endowments) along with parental (or other) characteristics to see how intergenerational genetic and environmental correlations may mediate each other. Furthermore, in both twin and adoptee study designs, *we cannot separate out genetic effects from prenatal environment.* In the case of adoptees, it may be that the important contributions of birth mothers are related to the uterine environment (including her diet and behavior during pregnancy) and not her genetic bequest. Such a possibility is raised by the robust literature showing that prenatal environment matters dearly to children’s development and ultimate socioeconomic success (see, e.g., Almond, Chay, and Lee 2005; Almond and Mazumder 2011; Black 2007; Conley and Bennett 2000; Torche and Echevarría 2011). Without a direct measure of genotype, extant research is not able to answer the question of whether observed associations across generations are largely social or biological in nature and whether social and genetic inheritances mediate or moderate each other’s influence.

Moderation of genotype by environment has long been an interest of social scientists. By way of example, Turkheimer et al. (2003) find that among low-income children, the heritability of IQ is lower than it is for higher-income children. Likewise, Guo and Stearns (2002) show that the heritability of IQ is lower for blacks than for whites. In both cases, the researchers interpret this to mean that environmental disadvantages—such as a lack of parental resources, poor schooling conditions, or simple racism—prevent the full realization of genetic potential. In other words, there is an implied conditionality such that potential intellectual ability is inherited but requires environmental conditions of human capital investment to be realized in the form of IQ (or educational attainment or income, for that matter; cf. Becker and Tomes 1994; Behrman, Pollak, and Taubman 1995; Behrman, Rosenzweig, and Taubman 1996). If such genotype-by-environment interaction effects hold true, this would augur policy interventions that target groups defined by social categories—socioeconomic status (SES) or race—to equalize genetic effects (i.e., level the playing field; Bearman 2013; Fletcher and Boardman 2013; Mitchell et al. 2013).^2^

The article proceeds as follows: Availing ourselves of two data sets with molecular genetic markers as well as educational information for parents and offspring (the Framingham Heart Study and the Health and Retirement Study), we first estimate overall latent heritability of education using an approach that relies on unrelated individuals (genomic-relatedness-matrix restricted maximum likelihood estimation [GREML]). We then utilize results from independent genome-wide studies of education to calculate a genomic risk score (GRS) for those two samples. Finally, we ascertain whether the inclusion of this genomic risk score substantially alters parameter estimates for parental education variables on offspring education and whether this genomic risk score moderates the effect of other, sociodemographic variables. In the following, we detail this novel approach to interrogating genetic mediation and moderation in models of educational inheritance.

## The Age of Molecular Markers

The recent collection of genetic markers from respondents of large and representative samples of adults has opened up an opportunity for researchers to directly confront and measure one of the two main “lurking” variables that threaten to bias traditional models of socioeconomic attainment (the other perhaps being the influence of cultural practices that are also transmitted across generations). Many novel approaches are possible as a result of the direct measurement of individual genetic variation. In the present study, we deploy two.

First, rather than fixing the values of genetic relatedness (e.g., 1 for monozygotic twins and 0.5 for dizygotic twins) in the twin based models, genome-wide similarity among biological siblings has provided comparable estimates of heritability without the strong assumptions that accompany the twin model (Visscher, Medland, and Ferriera 2006). More importantly, this same approach has been extended to pairs of unrelated persons in the population (Yang, Benyamin, and McEvoy 2010). Briefly, an estimate of genetic similarity is computed between any two individuals. This measure of identity by state (IBS) is then compared to phenotypic similarity of each pair of unrelated persons to estimate heritability. This approach, referred to as GREML, has been deployed for a variety of phenotypes, including height (Yang et al. 2010), schizophrenia (Purcell et al. 2009), smoking (Belsky et al. 2013a), asthma (Belsky et al. 2013c), body mass index (Belsky et al. 2012), educational attainment (Rietveld et al. 2013), and political and economic preferences (Benjamin et al. 2012).

The most important shortcoming of GREML estimates is that they still focus on a latent quantity (heritability) that cannot be used to directly test whether genotype mediates or moderates observed phenotypic relationships across generations. To do that, we need a measured—rather than latent—indicator of genotype. One method would be to test each measured allele separately, but this approach is seriously hobbled by the combination of weak effects of individual alleles on something as complex, distal, and polygenic as educational attainment. Instead, the solution at present is to collapse all the information from thousands or millions of markers into a single scalar that can then be easily deployed. This scale is known as a GRS and requires extremely large sample sizes that vastly exceed those available to any single social scientific study.

To address this sample size challenge, Rietveld et al. (2013) recently conducted a genome-wide association study of 126,559 individuals from 54 distinct cohorts to search for genetic variants that may be associated with educational attainment. Rietveld et al. conducted what is called a genome-wide association study (GWAS), an atheoretical approach to gene discovery where hundreds of thousands of single nucleotide polymorphisms (SNPs) are tested for association with an outcome of interest one by one using an ordinary least squares (OLS) regression framework. The GWAS focused on individuals of European descent to avoid issues related to population stratification—that is, nonrandom association of environments with genetic variation due to ancestry. Although three SNPs were identified as being significant (after correcting for multiple testing) and replicated in an independent sample, the greater significance of this study is that it allows for the construction of a polygenic risk score for educational attainment. A common approach to constructing such GRSs is to take a weighted sum of SNPs, where the weights are given by the estimated coefficients from the OLS regressions in the GWAS (For other examples of GRS deployment, see, e.g., Belsky et al. [2012], Belsky et al. [2013a], Belsky et al. [2013c], Benjamin et al. [2012], Purcell et al. [2009], Visscher, Yang, and Goddard [2010], Yang et al. [2010].) While only three alleles reached what statistical geneticists call genome-wide significance (*p* < 5 × 10^−8^) and replicated in the independent samples, these explained a trivial amount of the total variance in years of schooling or college attendance. Relaxing the significance threshold for SNPs included in the genetic risk score for educational attainment continually increases the predictive power. When all SNPs are taken into account, this single scalar can explain between 2 and 3 percent of the variance in years of schooling. This suggests that to the extent that it is associated with genotype, educational attainment—as we might expect—is driven by many small effects across the entire genome. This finding has further been replicated in new samples with stricter controls and the deployment of sibling fixed effects models (Rietveld et al. 2014). Furthermore, these risk scores have been shown to add predictive power over and above measured family history—at least in the health domain (Belsky, Moffitt, and Caspi, 2013b).

Two and a half percent is a relatively small contribution to our understanding of educational outcomes, especially when compared to the published meta-analyses that find that genetic factors account for up to 40 percent of the variation (Branigan et al. 2013). There are several important explanations for this so-called missing heritability (de Los Campos et al. 2013), including estimation error in the coefficients from the GWAS and sample size. With this caveat in mind, we turn to the aims of the present study. We build on Rietveld et al. (2013, 2014) by examining *intergenerational* models of educational attainment that include genetic endowment in both the parental and offspring generations. This is motivated by the assumption that if genetic factors mediate the relationship between parent and offspring educational attainment, then controlling for these genetic factors (via the polygenic risk score) should significantly lower the coefficient on parental education. This is a direct test of the genetic transmission hypothesis that improves on adoption and kinship studies, which may confound prenatal effects with genetic ones, as well as studies that control for IQ, which itself is affected by social environment (and may be partially endogenous to educational attainment itself).

We also investigate whether the effects of offspring genotype interact with the socioeconomic conditions of the parental household—specifically with maternal education. That said, an ideal test would be examine if the underlying genotype interacts with exogenous environmental shocks, such as school or tax policy interventions (Fletcher and Conley 2013). We know of no natural experiment that can be utilized within these data. Thus it remains possible that any significant interaction effects we discover are not true gene-by-environment effects but rather gene–gene interactions between measured genotype and unmeasured genotype of parents or the offspring. To mitigate this possibility, we also directly test for interaction effects between parental educational genotype and offspring educational genotype. Although this does not guarantee that our measured environmental variables are truly environmental (and not simply proxies for unmeasured genetic factors), such analysis should give us a sense of whether such confounding is likely to be driving our results.

## Data

The data for the present study come from the second- and third-generation respondents of the Framingham Heart Study (FHS) as well as from the Health and Retirement Study (HRS).We describe the specifics of the genotyping for each data set in Appendix A in the online supplement. We show descriptive statistics and baseline regression models compared to the white sample of the 2012 General Social Survey (GSS) for comparative purposes. The GSS is well known to social scientists and has been described extensively elsewhere (Davis and Smith 1992). We focus only on non-Hispanic whites for two reasons: FHS is a predominantly white sample to begin with, and more importantly, the polygenic risk score was obtained from a consortium that included only respondents of European heritage. Because there are different allele frequencies and greater genetic diversity among those of African descent (Tishkoff et al. 2009), it is challenging to develop polygenic scores for white, black, and Latino respondents that have the same measurement quality. Given this measurement issue in conjunction with the population differences in educational attainment, we chose to focus exclusively on a subsample of non-Hispanic and white respondents from both studies. Although it is not a goal of our current study, we encourage future researchers to extend our results via cross-ethnic replication. Our within-family models of full-sibling differences obviate any confounding of ethnic, cultural, or other inherited environmental forces, on one hand, and genotypic effects, on the other.

## Results

Table 1 shows descriptive statistics for these three samples. The most obvious difference between the populations is with respect to age. The GSS shows a mean age of roughly 50 years (49.05) with considerable variability (*SD* = 17.10 years). The third-generation respondents of the FHS who are included in our sample (i.e., have valid responses on both the social and genetic variables of interest as well as valid data on their parents) are just under a decade younger at 39.49 years of age on average, with concomitant lower variability as well (*SD* = 7.67 years). Meanwhile, the HRS sample is much older, by design, with a mean age of 68.17 years and a standard deviation of 10.5 years by virtue of its cohort design (we randomly select a wave for each respondent). While the age distribution does vary considerably, the sex ratio is almost the same, ranging from 50 to 58 percent female across the samples. Finally, the mean education levels also vary between the different studies for a variety of reasons, including attrition, region, age, and cohort effects. The FHS displays the highest mean education levels for both the respondents and their parents. This may be due to the fact that Massachusetts is a state with high average educational levels as compared to the nation writ large. For example, the mean rate of college graduation was highest in Massachusetts of all the states in the United States at 38.2 percent in 2009; meanwhile, the nation as a whole had a mean bachelor degree attainment rate of 27.9 percent (U.S. Census Bureau 2012). These state differences—along with cohort effects (namely, that the GSS sample is older)— probably account for the higher parental education levels in the FHS sample. Finally, due to their belonging to an older birth cohort, the HRS respondents have the lowest education levels, and their parents have completed just over 10 years of schooling on average. Because others have demonstrated differences in our ability to detect genetic associations for different birth cohorts (Boardman, Blalock, and Pampel 2010), it is important to consider these environmental and compositional differences in the two studies.

In Table 2, we show the bivariate relationships between our variables. In both samples, parental education is highly correlated among spouses (i.e., parents). In the FHS sample, we see that the spousal correlation among parents is 0.53; for HRS, it is even higher at 0.61. Moreover, in the FHS sample—for which we have parental genotype—the correlation of parental educational genetic risk scores is 0.22—that is almost half of the total phenotypic correlation in years of schooling between parents. This sizable genetic correlation in educational propensity stands in contrast to the much more modest share of educational assortative mating that has been associated with genome-wide (i.e., overall rather than specific) genetic correlation between spouses in two recent articles (cf. Domingue et al. 2014; Guo et al. 2014) and suggests that deployment of genetic risk scores may reveal genotypic bases of assortative mating that are not adequately captured by simple marker-by-marker analysis or by comparisons of overall genetic similarity. The correlation of parental genotypes has implications for heritability estimates from twin studies because random mating is a crucial assumption in such models used to estimate the genetic component of traits. Namely, positive assortative mating on the relevant genetic loci leads to downward bias in heritability estimates while negative assortative mating leads to upward bias.

While assortative genetic mating affects latent estimation of heritability using twin or extended twin designs, it should not affect GREML, which focuses on unrelated individuals. In Table 3, we present GREML estimates of heritability for the two samples to provide a benchmark against which to measure the effect of the genomic risk score we have computed. GREML estimates pairwise genome-wide relatedness values are calculated; as is standard in the literature, only pairs that are less than 2.5 percent related are retained, to eliminate any cryptically related pairs from the sample. Using this estimated measure of relatedness, genetic similarity can be compared to phenotypic similarity (in this case, years of schooling). The GREML heritability estimate is a proportion of the total variance accounted for by the genetic variance based on the genotyped SNPs. These results are shown in bold. For the FHS sample, we estimate a high but noisy heritability of 0.54. For the HRS, which has a much bigger sample size, the estimate is more precise and lower at 0.20. Both of these estimates are statistically significantly different from zero and fall well within the distribution of heritabilities found by Branigan et al. (2013) in their meta-analysis of twin-based estimates. Because these heritabilities are unaffected by nonrandom mating at the parental level, we use them as our benchmarks against which we assess the predictive power of the measured genotype (GRS) we construct for each sample.

We now turn to the central question of the article: to what extent do measured parent–child correlations in educational attainment reflect genetic inheritance? In model 1 for FHS in Table 4, we can see that each additional year of maternal education is associated with approximately 0.35 years of additional schooling among third-generation white FHS respondents. Model 2 demonstrates the effect of the polygenic risk score as calculated by Rietveld et al. (2013) and applied to the present FHS sample. Without age and sex controls, the GRS alone produces an *R*^2^ of 0.019, similar to the result reported by Rietveld et al.. Model 3 presents the key test for our question of whether the omission of genotype leads to specification error in the estimation of the effect of maternal education on offspring education. When we include both mother’s education and offspring’s genotype, we find that both are significant predictors of offspring education. While the point estimate for the genetic score of the respondent drops in magnitude significantly (by 55 percent) with the inclusion of maternal education as a measure (suggesting that some of the “genetic” effect may be due to its correlation with maternal education), we do not find that the parameter estimate for maternal education drops substantially—only 3.33%. If the proper scaling factor is 5 (i.e., the true heritability is ~20 percent, as it is for the more precisely estimated HRS sample and other samples used by prior research [Rietveld et al. 2013], and shared genetic similarity of parent and child is 50 percent), then one-sixth of the observed intergenerational correlation is due to genes and five-sixths is due to environmental influences (among whites in the FHS). We return to this calculation in the discussion section.

For now, we turn to the question of whether maternal genotype rather than offspring genotype is the key omitted variable. To test this possibility, we add parental genotype to the model to see if it has a direct effect on offspring educational attainment alone as well as net of parental education and offspring genotype and/or whether it explains part of the effect of parental education. In model 4 of the FHS data in Table 4, we show that the mother’s genetic risk score predicts offspring education when we only control for sex and age (or without those controls). The *R*^2^ for the model with only maternal risk score and no other controls is 0.008, less than half that of the offspring genetic risk score. However, it is worth noting that in models 5 and 6, we find no evidence that, net of observed maternal education (and/or offspring genotype), maternal genotype matters.

When we perform a similar exercise with data from the HRS, we find that a corroborating story emerges. In this data set, we do not have the genotypes of the parents, so we can only answer the question of whether offspring genotype biases or is biased by observed maternal education. The story that emerges is much the same. Both maternal education (model 1) and respondent genotype (model 2) predict offspring education when run separately. In model 1, each additional year of maternal schooling results in three-tenths of an additional year of school for the offspring. Likewise, in model 2, we see that a 1 standard deviation change in genetic score of the offspring is associated with 0.41 additional years of schooling. The score is more predictive for the HRS than for the FHS sample (*R*^2^ = 0.026). Despite a slightly larger effect for genotype and a slightly smaller effect for maternal education, when we compare across models in the HRS, the story is much the same as it was for the FHS sample. Namely, comparing models 2 and 3, we see that the coefficient for genotype drops by about a third in magnitude when maternal education is held constant—a slightly smaller drop than seen for the FHS data. Likewise, when we compare the coefficients for maternal education in model 1 to those with GRS controlled for in model 3, we find that it has dropped by 3.8 percent. Here, if we scale up to 20 percent heritability (a factor of 7.58 given the score’s *R*^2^ when run alone) and 50 percent relatedness, this would mean 14.4 percent of the intergenerational association between one parent and her offspring in educational attainment can be accounted for by genetic factors.

In Table 5, we test the Turkheimer hypothesis that genetic endowment interacts with parental SES—in this case, maternal education—such that among those offspring from lower-SES families, the effect of genotype is muted. Column 1 shows the baseline model reproduced from Table 2. Column 2 reports estimates with an interaction effect between maternal education and offspring genotype. This interaction term is not significant for either the FHS or HRS samples. It is, of course, possible that we are underpowered to detect an effect that is present, especially if the moderated effect is small in magnitude. However, we do not see any evidence of large interaction effects, as has been claimed in prior, twin-based studies.^3^

For model 3 of the FHS data, we estimate a parental genotype–offspring genotype interaction effect directly. Parental genotype is the latent lurking variable in prior studies that claim gene–environment interaction effects between genotype and social class (Turkheimer et al. 2003); therefore this is an important test in conjunction with our null result in column 3. And indeed, here we find that the only significant interaction effect in all the models is between maternal genotype and offspring genotype. This suggests that growing up with a genetically advantaged (or disadvantaged) mother enhances the effects of one’s own standing in the genetic lottery. The coefficient is positive such that a child born to a genetically average mother who, by the luck of recombination, has a genotype that is 1 standard deviation above the mean for the offspring generation will complete on average 0.10 more years of schooling than the child at the mean of the genotype distribution. However, if that same child was born to a mother who herself was also 1 standard deviation above the mean in the genetic lottery, the child’s advantage would be more than a quarter year of schooling. (If we control for the main effect of maternal GRS in this model, it is not significant, and the strength of the interaction effect actually increases from 0.15 to 0.16; results are available upon request from the authors.) Note that this is net of how many years of schooling the mother actually completed; that is, it is likely an interaction with her native cognitive or noncognitive ability (which itself predicts education), not her achieved educational (social) status. This result (though not reproducible in HRS without parental genotype data) suggests that there is a positive development feedback loop created when a gifted child has a gifted mother with whom to interact, independent of her actual educational attainment.

Finally, it could be the case that alleles are nonrandomly distributed across social groups, making the genetic effect spurious. For example, imagine group A has higher education on average than group B for historical, cultural, or economic reasons. Meanwhile, group A also scores higher on the polygenic risk score for education for random reasons of genetic drift. It could appear that the polygenic risk score causes educational attainment when it is really just acting as a proxy for socially observable differences. To address this concern, we run sibling fixed effects models. By comparing full siblings from the same family, concerns about genetic–environmental confounding are obviated because the differences in polygenic risk score between siblings stem wholly from random processes in reproduction. We do not have sibling data in the HRS, so we confine this analysis to the FHS sample. In Table 6, we break the overall regression out into within- and between-family components.^4^ When we add the risk score to a within-family model in column 1, it is significant and adds 1.24 percentage points of explained variation to the *R*^2^. When we confine estimation of the effect of the GRS to the between-family component in model 2, we find that the *R*^2^ for the score alone is 2.29 percent. This finding is due to the fact that because there is nonrandom mating at the parental level, the sibling correlation is higher than 0.5 (i.e., there is more variance in GRS across families than within them).

A different story emerges when we look at the effect of the coefficient itself. Here we find that the point estimate from the within-family regression is larger than in the between-family regressions. Within families, we see that for each additional standard deviation in GRS relative to one’s sibling, the educationally more endowed sibling completes 0.32 more years of schooling. But comparing unrelated individuals across families, the difference in schooling is only 0.27 years. In other words, it may be the case that any reinforcing effect on social stratification that the between-family distribution of genetic ability has on the reproduction of educational attainment across generations due to genetic assortative mating is compensated for by the greater effect of genetic endowment on schooling within families (i.e., between siblings) (cf. Domingue et al. 2014). How can we understand this greater effect within families? It may be the result of niche formation. Observed differences among siblings in ability may lead parents to reinforce those differences (Conley 2004). Alternatively, siblings themselves may pursue a strategy of differentiation that leads to an accentuation of genetic differences. Indeed, the sibling intraclass correlation in the FHS data is lower for education (0.43) than it is for the genetic risk score (0.59).

Finally, as a robustness check, in Table 7, we run the same models as those presented Table 4, but include father’s information in addition to mother’s. When we include father’s GRS in the FHS sample, where it is available, it is not significant (nor is mother’s GRS in this model). Likewise, the coefficient on father’s actual educational attainment on offspring educational attainment rises (although negligibly) when offspring, maternal, and paternal genotypes are held constant. In the HRS sample, the effect of paternal education drops slightly (in line with the drop to maternal education) when offspring genotype is held constant. In sum, the confounding of paternal education by off (Goldberger 1978) spring genotype is comparable to the same pattern as maternal education.

## Discussion

We have argued here that knowing the extent to which an outcome is associated with genotypes (i.e., heritability) does—contrary to Goldberger and Manski’s (1995) blanket negation—have social and policy implications. First, to the extent that an important outcome is related to a measureable genotype, we can know how to target interventions more precisely. If we wish to reduce inequality in educational outcomes, knowing individuals’ genetic propensity for educational attainment may help identify populations that are more or less in need of schooling interventions.^5^( Second, to the extent that an outcome is related to genotypes, this implies that there may be trade-offs between efficiency-maximizing policies and equality-promoting ones under the present societal equilibrium [Heath et al. 1985].) Lastly, and most relevant to the present analysis, the extent to which a phenotype is the result of a genotype has policy implications. If the observed effect of parental education on offspring schooling is largely due to intergenerationally correlated environments, then altering the distribution of education in the parental generation will also affect the distribution in the filial generation. However, if the intergenerational association is due to genetic characteristics, then even totally equalizing education in a given generation will have little effect on the next generation.

With these rationales in mind, we are the first researchers to deploy a polygenic risk score to model the transmission of educational attainment from parents to children. This approach avoids the confounding of genetic effects with prenatal environmental effects from which adoption studies suffer. Also, this approach complements twin models, which suffer from limits with respect to external validity and may confound exogenous pre- or postnatal environmental factors that covary with genetic relatedness. Finally, deploying explicitly measured genotype in models allows us to do a formal mediation analysis that is not possible in the variance portioning exercises inherent to the twin or adoption studies. By comparing across models of intergenerational educational inheritance with and without genotype controlled for, we are able to come to some tentative conclusions. First, when we include offspring genotype to control for spurious effects of parental education due to parent–offspring similarity in genetic educational endowment, we find in two different data sets that the coefficient for maternal education (our focus) or paternal education (adjunct analysis) is indeed slightly attenuated. The amount of attenuation is trivial, and a Hausman test reveals it not to be significantly different from zero. However, if we accept the point estimates as accurate and make a few other assumptions (see Appendix B in the online supplement for a thorough discussion of these), such as an overall heritability of education of 0.2 and that parents and offspring share half of their genes, then we can say that our best estimate is that one-sixth of the observed, raw mother–child correlation in education is due to genetic transmission. This is not a trivial amount of misspecification in social science models, nor is it a fatal amount.

Furthermore, because almost half of the variation in the polygenic risk score is within families, and the effect of this measure of “innate” ability is almost as predictive within families as between them (and because the magnitude of the parameter estimate is actually larger for within-family models), genetic stratification is not an accurate description of the interplay between genes and social status. *The Bell Curve* argues that meritocracy and assortative mating lead to a system of class stratification that is based on innate (i.e., genetic) endowment (Herrnstein and Murray 1994). If this were true, social policy to promote equal opportunity would be counterproductive—at least on efficiency grounds—because each individual will have reached the level of social status best suited to the individual’s native abilities. Meanwhile, by selectively breeding with others of similar genetic stock, parents would reinforce their offsprings’ advantages or disadvantages. Putting aside the weakness of their empirical analysis, such a putative vision would call into question the notion that intergenerational correlations in SES variables—such as income, occupation, or education—reflect a *lack* of meritocratic openness in a given society.

However, in our analysis, we find that despite a moderate degree of assortative mating on educational genotype (*r* = 0.22 among FHS parents and 0.22 among HRS spouses), the within-family differences in genetic stock that generate observed social mobility (i.e., sibling or parent–child differences) partially counteract any between-family effects of the distribution of genetic stock, educational attainment, and assortative mating at the parental level. While assortative mating means that there is indeed greater variance between families on genotype than within families (the sibling *r* in FHS is 0.59), the actual strength of the effect (which is greater within families) may be due to the fact that small differences in endowment between siblings generate a very elastic social response of differentiation within the household. That is, siblings may engage in niche formation that accentuates the differences in genotype, or it may be the case that parents differentially invest and create larger differences as the endowments of their various offspring are revealed to them through interaction over the course of childhood. In other words, the randomness of recombination and segregation of alleles during sexual reproduction along with the within-family accentuation of endowment effects serve to help counteract any cross-familial social sorting by genotype to help “reset” the genetic playing field for the next generation.

Lastly, parental genotype has no net effect on offspring educational attainment once genetic transmission (i.e., offspring genotype) and parental years of schooling are controlled for. That is, parents’ genes are not leading to social advantages above and beyond their own schooling. The counterfactual of genetically advantaged parents having *not* attained schooling themselves being able to nonetheless pass on educational advantages to their children culturally (i.e., above the abilities they pass on genetically) does not appear to hold true. An important caveat to all this analysis is that heritability—that is, genetic effects—is, of course, highly contingent on social structure, whether measured latently through kin correlations or directly by a GRS. Indeed, heritability is not a fixed parameter across time and place but is always a “local perturbation analysis” estimate, as cogently argued by Feldman and Lewontin (1975:1163) 35 years ago. That said, we still believe it is useful to understand the relationship between genotype and phenotype even in partial equilibrium when it comes to important social outcomes like educational attainment—even if only to know how biased our measured relationships among putatively social variables are. Our estimates then become the fodder for future analysis of waning or waxing articulation between social and genetic reproduction.

## Supplementary Material

Appendix A and B

## Figures and Tables

**Table 1:** Descriptives for Variables Used in Analysis by Sample

	2012 GSS		FHS		HRS	
	Mean	SD	Mean	SD	Mean	SD
Resp. highest grade completed	14.25	2.79	15.08	2.06	13.42	2.46
Mother’s highest grade completed	12.29	3.12	13.66	2.26	10.40	3.01
Father’s highest grade completed*	12.23	3.74	14.41	3.04	10.02	3.46
Female sex	0.54	0.50	0.50	0.50	0.58	0.49
Age	49.05	17.1	39.49	7.67	68.17	10.50
Year of survey					2, 006.90	2.20
Raw educational polygenic risk score			−9.70E-06	7.21E-06	1.49E-06	7.68E-06
Raw maternal educ. polyg. risk score			−9.89E-06	7.51E-06		
Raw paternal educ. polyg. risk score†			−9.65E-06	7.12E-06		
*N*	1,052		968		6,186	
Number of families	1,052		460		4,867	

* *N* for this variable in the HRS is only 5,807.

† *N* for FHS for this variable is 741 individuals from 241 families.

**Table 2:** Correlations among Genetic and Education Variables in FHS & HRS Samples

Framingham Heart Study (N = 741)	Resp. Educ.	Mother Educ.	Father Educ.	Resp. Score	Mother Score
Respondent’s highest grade completed	1				
Mother’s highest grade completed	0.35	1			
Father’s highest grade completed	0.32	0.53	1		
Respondent’s educational genetic score, standardized	0.16	0.24	0.13	1	
Mother’s educational genetic score, standardized	0.08	0.24	0.15	0.59	1
Father’s educational genetic score, standardized	0.11	0.16	0.09	0.60	0.22
Health and Retirement Study (N = 6,186)					

Respondent’s highest grade completed	1				
Mother’s highest grade completed	0.37	1			
Father’s highest grade completed	0.38	0.61	1		
Respondent’s educational genetic score, standardized	0.17	0.08	0.08	1	

**Table 3:** Genomic-Relatedness-Matrix Restricted Maximum Likelihood Estimation (GREML)-based Estimates of the Heritability of Years of Schooling, by Sample

Framingham Heart Study (N = 932)	
V(G): Variance in genotype	3.81 (1.68)
V(e): Residual error	3.28 (1.64)
V(P): Variance in phenotype	7.09 (0.33)
V(G)/V(P)	**0.54** (0.23)
logL	−1, 377.16
logL0	−1, 382.52
Likelihood Ratio Test	10.73
p-value (df = 1)	0.001
Health and Retirement Study (N = 6,186))	

V(G): Variance in genotype	1.20 (0.59)
V(e): Residual error	4.90 (0.49)
V(P): Variance in phenotype	6.10 (0.16)
V(G)/V(P)	**0.20** (0.09)
logL	−7, 700.68
logL0	−7, 703.49
Likelihood Ratio Test	5.62
p-value (df =1)	0.009

*Note:* Standard errors in parentheses.

**Table 4:** Regression Models of Respondent’s Total Years of Completed Education with Standard Errors Robust to Clustering on Family ID, by Sample

Framingham Heart Study	(1)	(2)	(3)	(4)	(5)	(6)
Female sex	0.36† (0.12)	0.38† (0.13)	0.35† (0.12)	0.38† (0.13)	0.35† (0.12)	0.35† (0.12)
Age	0.01 (0.01)	−0.01 (0.01)	0.01 (0.01)	−0.01 (0.01)	0.01 (0.01)	0.01 (0.01)
Mother’s highest grade completed	0.35† (0.03)		0.34† (0.03)		0.35† (0.03)	0.34† (0.03)
Respondent’s educ. genetic score, std.		0.27† (0.07)	0.12* (0.07)			0.16* (0.08)
Mother’s educ. genetic score, std.				0.17* (0.08)	0.02 (0.07)	−0.06 (0.09)
Constant	9.34† (0.62)	14.88† (0.45)	9.50† (0.63)	14.88† (0.44)	9.37† (0.64)	9.48† (0.64)
*R*^2^	0.14	0.03	0.15	0.02	0.14	0.15
*R*^2^ for score w/out other controls		0.02		0.01		
Health and Retirement Study	(1)	(2)	(3)			

Female sex	−0.32† (0.06)	−0.40† (0.03)	−0.30† (0.06)			
Age	−0.01* (0.00)	−0.04† (0.00)	−0.01† (0.00)			
Survey year	0.02 (0.01)	0.00 (0.01)	0.02 (0.01)			
Mother’s highest grade completed	0.30† (0.01)		0.28† (0.01)			
Resp. educational genetic score, std.		0.41† (0.03)	0.33† (0.03)			
Constant	−34.61 (26.71)	−84.04† (28.04)	−43.16 (26.44)			
*R*^2^	0.14	0.05	0.16			
*R*^2^ for score w/out other controls		0.03				

Note:

* *p* < 0.05;

† *p* < 0.01.

Framingham Heart Study N=968; 460 Families. Health and Retirement Study N=6,186; 4,867 Families.

**Table 5:** OLS Regression Models of Respondent’s Total Years of Completed Education with Interaction Effects, with Standard Errors Robust to Clustering on Family ID, by Sample

Framingham Heart Study	(1)	(2)	(3)
Female sex	0.35† (0.12)	0.35† (0.12)	0.37† (0.12)
Age	0.01 (0.01)	0.01 (0.01)	0.01 (0.01)
Mother’s highest grade completed	0.34† (0.03)	0.34† (0.03)	0.34† (0.03)
Respondent’s educational genetic score, standardized	0.12* (0.07)	−0.28 (0.46)	0.10 (0.06)
Respondent’s educ. genetic score * Mother’s highest grade completed		0.03 (0.03)	
Respondent’s educ. genetic score * Mother’s educ. genetic score, std.			0.15† (0.05)
Constant	9.34† (0.62)	9.55† (0.63)	9.32† (0.64)
*R*^2^	0.14	0.15	0.15
Health and Retirement Study	(1)	(2)	

Female sex	−0.30† (0.06)	−0.30† (0.06)	
Age	0.01 (0.03)	−0.01† (0.03)	
Survey year	0.03* (0.01)	0.03* (0.01)	
Mother’s highest grade completed	0.28† (0.01)	0.28† (0.01)	
Respondent’s educational genetic score, standardized	0.33† (0.03)	0.27† (0.10)	
Respondent’s educ. genetic score * Mother’s highest grade completed		0.01 (0.01)	
Constant	−34.61 (26.71)	−43.60 (26.45)	
*R*^2^	0.14	0.16	

* *p* < 0.05;

† *p* < 0.01.

Framingham Heart Study N=968; 460 Families. Health and Retirement Study N=6,186; 4,867 Families.

**Table 6:** Within-Family, Between-Family Regression Models of Respondent’s Total Years of Completed Education with Standard Errors Robust to Clustering on Family ID

	(1) Within	(2) Between	(3) Total
Female sex	0.44† (0.15)	0.41* (0.20)	0.38† (0.13)
Age	0.04 (0.02)	−0.01 (0.01)	−0.01 (0.01)
Respondent’s educational genetic score, standardized	0.32† (0.11)	0.27† (0.09)	0.27† (0.07)
Constant	12.90† (0.85)	15.06† (0.90)	14.88† (0.45)
*R*^2^	0.04	0.04	0.03
*R*^2^ for score w/out other controls	0.01	0.02	0.02

Note

* *p* < 0.05;

† *p* < 0.01. Framingham Heart Study N=968; 460 Families.

**Table 7:** OLS Regression Models of Respondent’s Total Years of Completed Education with Standard Errors Robust to Clustering on Family ID, by Sample; Fathers’ Characteristics Included

	FHS		HRS	
	(1)	(2)	(3)	(4)
Female sex	0.55† (0.14)	0.53† (0.14)	−0.33† (0.06)	−0.31† (0.06)
Age	0.01 (0.01)	0.01 (0.01)	0.00 (0.00)	0.00 (0.00)
Survey year			0.02 (0.01)	0.02 (0.01)
Mother’s highest grade completed	0.25† (0.04)	0.24† (0.04)	0.17† (0.01)	0.17† (0.01)
Respondent’s educational genetic score, standardized		0.24* (0.11)		0.32† (0.03)
Mother’s educational genetic score, standardized		−0.14 (0.11)		
Father’s highest grade completed	0.13† (0.04)	0.13† (0.04)	0.17† (0.01)	0.17† (0.01)
Father’s educational genetic score, standardized		0.00 (0.09)		
Constant	8.45 (0.74)†	8.65† (0.90)	−26.95 (26.80)	−35.35 (26.54)
*R*^2^	0.16	0.17	0.18	0.20

Note:

* *p* < 0.05;

† *p* < 0.01.

Framingham Heart Study N=741; 358 Families. Health and Retirement Survey N=5,807; 4,619 Families.

## Footnotes

1 However, this line of argument conflates genetics with merit. For example, if social sorting in the educational system (or labor market) took place based on eye color, it would be close to 100 percent heritable/genetic, yet few would argue that this form of assignment would be meritocratic, because meritocracy also assumes a legitimacy to the characteristics by which we sort individuals into roles—not merely a biological or natural basis to those characteristics. Admitting students to the University of California, Berkeley based on basketball shots would be fair—in the sense that everyone might know the rules beforehand and be subject to the same constraints in a task that is easily observable with minimal measurement error—but few would agree that this would be meritocratic, because there would be a mismatch between the institutional raison d’être of the university as an institution and the sorting mechanism.

2 However, if we ascertain genetic influence (i.e., heritability) latently through twin comparisons (as prior scholars largely do), it is difficult to know if a reduced or enhanced heritability for a given group is due to differences in (1) differential effects of prenatal conditions (cf. Conley and Strully 2012) or (2) differences in the level of variation in genotype (numerator) or the denominator (phenotype), or (3) whether, instead, it is truly a difference only in the covariance between genotype and phenotype by subgroup. However, if we measure genotype directly—as we do here—we can interrogate the distribution (i.e., variance) of genotypic propensity toward educational attainment by subgroup and the variance in phenotype by subgroup, and we can estimate interaction effects between genotype and subgroup (i.e., test whether the covariance between genotype and phenotype differs). Thus here we provide a much more direct test as to whether the “natural” genetic tendencies of one group—say, women or those from low-SES families—are repressed by social structure.

3 Of course, had we found a significant interaction with maternal education, it could have been the case that this putatively environmental measure we are interrogating was actually acting as a proxy for unmeasured parental genotype. In an ideal world, we would have an exogenous environmental source of variation in parental education and would interact offspring genotype with this instrumented measure of parental education. Of course, it is hard to envision what such an instrumental variable would be that would not violate the exclusion restriction (i.e., have no direct effect on offspring education other than through years of schooling of the parent). In lieu of this idealized study design, we control for maternal genotype in our models and do not find a significant offspring genotype–parental phenotype interaction. If we had found a significant interaction effect, endogeneity concerns would warrant caution in interpreting this as a true gene–environment interaction. Given our finding is in favor of the null hypothesis, such concerns are secondary.

4 In the FHS sample, we see that overall, family background explains ~43 percent of the variation in educational attainment—a figure that corresponds to what others have found (Conley and Glauber 2008; Hauser, Sheridan, and Warren 1999; Kuo and Hauser 1995; Olneck 1976; Teachman 1987; Warren, Sheridan, and Hauser 2002).

5 Of course, how responsive a given genotype is to educational interventions—that is, the returns to human capital investments—is a different question than how predicted education is by a linear genotypic measure.
