# Supplementary material for: Is the Effect of Parental Education on Offspring Biased or Moderated by Genotype?
Source: Sociol Sci. Author manuscript; Available in PMC 2017 Oct 17. (PMC5644503; doi:10.15195/v2.a6)
Supplement: Appendix A and B [file NIHMS861959-supplement-Appendix_A_and_B.pdf]

Appendix A: Genetic Data Description

Genotypes for the FHS were assayed using the Affymetrix GeneChip Human Mapping 500K Array and the 50K Human Gene Focused Panel. Genotypes were determined using the BRLMM algorithm (additional details can be found at Jaquish, 2007). Of the original 500,568 SNPs, 260,469 were left after cleaning (e.g., HWE screens and a MAF cutoff of 0.05). The screens were conducted using all available individuals with genetic data, not only those who were included in this analysis. Individuals were considered outlying from European descent if one or more of the first four eigenvectors was more than three standard deviations removed from the mean. SNPs with minor allele frequency (MAF) < 0.01, call rate < 0.95, or Hardy–Weinberg equilibrium test p-value < 0.001 were removed.

Genotypic data for the HRS sample were obtained for 12,507 study subjects by the Illumina HumanOmni2.5-4v1 array. According to HRS documentation, "the median call rate was 99.7% and the error rate estimated from 336 pairs of study sample duplicates is 6×10^{-5}." Samples with an overall missing call rate > 2% were removed. For additional details on quality control of HRS genetic data, please see http://hrsonline.isr.umich.edu/sitedocs/genetics/HRS_QC_REPORT_MAR2012.pdf. Including respondents with valid information on parental education and own education yielded a sample of 6,186 individuals from 4,867 households (there are 2 individuals from some households because both spouses are sampled if living).

Appendix B:

Description of Underlying Assumptions about the Genotype-to-Heritability Ratio

We assume that parents' genotypes and offspring genotypes are correlated at 0.5 (Jones and Martin 1992; Weir et al. 2006). From the GREML analysis of the well-powered HRS data set, we assume that offspring genotype is correlated with offspring phenotype at 0.2. Finally, from our data, we assume that parental phenotype is correlated with offspring phenotype at ~0.35. In this scenario, our prior is that just under one-third the parent–child correlation in education is due to their genetic covariance since the product of the explained variance by genotype and the genetic relatedness coefficient is 0.1 (i.e., 0.5 times 0.2).

However, with the educational genetic risk scores we calculate, we only measure approximately two percentage points of the 20 percent of educational-genotypic covariance, so the question becomes whether the 2 percent explained by our GRS is systematically biased with respect to the underlying, putative 10 percent of educational variance that is associated with offspring genotype, on one hand, and parental education, on the other hand. That is, if the missing 18 percent of genotype–education covariance displays the same covariance with parental education as our observed genotype–parental education covariance, then the fact that we are measuring only 2 percent out of 20 percent merely implies that as we obtained a better measure of genotype, we would increase in a linear fashion the degree to which our observed measure of genotype mediates the parent–offspring educational correlation. Assuming that this is the case, with increased power and better measurement of the causal alleles, such attenuation bias in the predictive power of the genetic score would decline and the

three-way relationship between child-measured genotype, parental education, and offspring education would increase. In this case of random sampling error and measurement error being the problem behind the "missing heritability" in our sample, our inferences are still unbiased, and we can extrapolate that at a maximum, offspring genotype mediates ~5 times the observed mediation in our models when we include our GRS as a mediator of parental education.

However, if it is the case that the measured component of the latent variable "education genotype" (G_c) is related to the covariance of parental and offspring education in a way that differed from the unobserved component, then our inferences from the present analyses would be flawed. This could be the case if, for instance, a significant portion of the relationship between child genotype and phenotype were due to new mutations in the germline that, therefore, manifest in the offspring but not the parents. This is a mechanism that has been suggested to explain the rising prevalence of autism spectrum disorders, for instance (Liu et al. 2010). In this scenario, it is highly unlikely that such novel mutations would be picked up by the existing genotyping platforms or even the imputation approaches to haplotype databases such as 1,000 genomes or HapMap (Gibbs et al. 2003; Kuehn 2008; SEQUENCING 2007; Siva 2008; Thorisson and Smith 2005; Via et al. 2010), thus they would not be in the explained 2 percent but would be in the missing heritability. However, they would be orthogonal to parental education, since they were not shared by that generation. Such a dynamic would imply that simply inflating our observed estimate of mediation by fivefold would result in an overestimation of the extent to which offspring genotype is biasing parent–offspring education models.

Another dynamic that could cause the fivefold figure to be an overestimate is if cross-allelic interactions were important drivers of the genotype-education correlation. Namely, imagine a situation where two biallelic loci influence education (and the two alleles at each locus) each have a population frequency of 50 percent. In scenario 1, the two loci have purely additive, independent effects. In this case, each parent should pass on 50 percent of its genotype–education covariance to the offspring so that half of the genetically explained variation of offspring education should overlap with the parents—that is, if we think the genetic proportion of the variance in education is ~20 percent, then genotype should account for 10 percent of the observed parent–child correlation in phenotype. However, now envision the scenario where the combination of alleles at loci A and B interact to produce educational outcomes—that is, their effects are not independent. In this case, now only 12.5 percent of the ~20 percent genotypically explained variance falls jointly within the shared region of the parent–offspring phenotypic correlation. If we posit higher-order interaction effects, it is possible to envision worlds where in the cross section genetics still matter greatly (i.e., the putative 20 percent we estimate here) in explaining the distribution of years of schooling, but the proportion of observed parent–child phenotypic covariance explained by shared parent–child genotypic covariance approaches nil. Indeed, with only ~20,000 genes and a highly embedded network of known gene–gene interactions, cross-locus effects are almost certain to play an important role in any complex, quantitative phenotype (such as educational attainment) (Conley 2009). The only problem with this scenario from the point of view of our estimation procedure is that by virtue of constructing a linearly additive GRS, the proportion of variation that we explain by definition excludes cross-allelic interaction effects. Thus, the more likely scenario is where our measured

educational genetic score overlaps to a greater degree with the shared parent–child component of education than it would if it were randomly sampled from the 10 percent of shared parent–child genotype–education covariance. This is true because we know that the parent–offspring correlation in this linear predictor is ~0.5 (it is actually 0.59 in the one data set with genetic scores for both generations [FHS]), and any multiplicative index of genotype would be correlated at less than 0.5. (The higher than 0.5 score is due to the fact that parents' scores are correlated at 0.22; i.e., there is nonrandom mating.)
